# Perceived feasibility of peer-delivered mental health and psychosocial support interventions for people living with HIV/AIDS at Ryan White-funded clinics in the southeastern United States: a Consolidated Framework for Implementation Research study of health care worker perspectives

**DOI:** 10.3389/frhs.2026.1896794

**Published:** 2026-08-11

**Authors:** Caroline W. Kokubun, Sophia C. Garbarino, Celeste K. Ellison, Benjamin G. Druss, Briana A. Woods-Jaeger, Ameeta S. Kalokhe, Jessica M. Sales

**Affiliations:** 1 Department of Behavioral, Social, and Health Education Sciences, Rollins School of Public Health, Emory University, Atlanta, GA, United States; 2 Department of Health Policy and Management, Rollins School of Public Health, Emory University, Atlanta, GA, United States; 3 Hubert Department of Global Health, Rollins School of Public Health, Emory University, Atlanta, GA, United States; 4 Division of Infectious Diseases, Emory University School of Medicine, Emory University, Atlanta, GA, United States

**Keywords:** HIV/AIDS, interventions, mental health, peer support, task shifting, task sharing, trauma

## Abstract

**Background:**

People living with HIV/AIDS and violence-related trauma histories (PLWH/T) may have unmet mental health needs due to high rates of mental disorders coupled with mental health provider shortages, as well as other barriers to care. Our prior work with PLWH/T in Metro Atlanta revealed a desire for peer-delivered mental health and psychosocial support (MHPSS) services, such as peer mentorship/navigation, peer support groups, cognitive behavioral therapy, and Psychological First Aid/Mental Health First Aid.

**Methods:**

In the present qualitative study, we assessed the feasibility of implementing or expanding these interventions through interviews with health care workers (HCWs) at Ryan White-funded clinics (RWCs) using the Consolidated Framework for Implementation Research to guide data collection and analysis.

**Results:**

Key barriers included structural characteristics (i.e., staffing limitations), lack of available resources (e.g., funding), and engaging innovation deliverers and innovation recipients. Key facilitators included financing (e.g., grants), need, and access to knowledge & information (e.g., training).

**Discussion:**

Overall, RWC HCWs perceived these interventions to be feasible to implement/expand. Future research should assess cost-effectiveness and explore pathways of mobilizing peers to deliver MHPSS interventions to PLWH/T.

## Introduction

People living with HIV/AIDS and violence-related trauma histories (PLWH/T) are disproportionately more likely than the general population to experience mental health conditions, such as posttraumatic stress disorder (PTSD), depression, and anxiety (1). The southern United States (U.S.) is the epicenter of the nation's HIV epidemic (2); however, PLWH/T in this region are less likely to utilize mental health and psychosocial support (MHPSS) services, particularly in rural areas, due to less availability and a shortage of mental health professionals (3–5). Additionally, PLWH/T may experience challenges accessing MHPSS services due to structural barriers (e.g., lack of transportation, high costs, insufficient insurance coverage) (6), and other factors, such as mental health stigma, under-recognition of symptoms, and concerns about privacy and confidentiality (7). PLWH/T and their health care providers must also contend with the intersection of poverty and interpersonal-level factors such as racism, discrimination, and lack of social support/isolation that affect PLWH and further complicate their treatment (8, 9).

In a recent qualitative study to assess the acceptability of implementing violence screenings and support practices in Ryan White-funded clinics (RWCs; medical safety-net clinics serving PLWH in the U.S.) by Kalokhe et al. (10), MHPSS services were reported as needed and desired by PLWH/T in Metro Atlanta. Subsequently, focus groups with PLWH/T in Metro Atlanta were conducted to assess the acceptability of peer-delivered MHPSS interventions, i.e., cognitive behavioral therapy (CBT), Psychological First Aid (PFA)/Mental Health First Aid (MHFA), peer mentorship/navigation, and peer support groups. Results indicated that focus groups found peer support interventions to be acceptable and responded favorably to the concept of peer-delivered CBT and PFA/MHFA.^1^

Evidence from both mental health and HIV intervention literature supports the implementation of task shifting and sharing (e.g., mobilizing lay health workers, such as peers) to deliver MHPSS interventions to PLWH/T, with studies indicating that peer-delivered task shifting and sharing interventions are both acceptable and feasible to people in need of mental health services (11–13), as well as PLWH (14–22). In low- and middle-income countries (LMICs), peer support has been effective in reducing symptoms of depression and anxiety among PLWH (23–25); however, little is known about peer support interventions that are intended to address mental health disorders among PLWH in high-income countries (HICs), such as the U.S. (24). Additionally, a scoping review of MHPSS task shifting and sharing interventions for PLWH conducted by Kokubun et al. (26) found that interventions that seek to address trauma among PLWH remain understudied globally, continuing a troubling pattern first identified by LeGrand et al. (1).

Although peer support interventions are not new to HIV care settings and have been used to promote continuity of HIV care, less is known about their application in promoting client mental health and engagement in MHPSS services, and whether such adaptations of the intervention are feasible. However, a few studies have previously applied the Consolidated Framework for Implementation Research (CFIR) by Damschroder et al. (27) to the implementation of peer support in mental health care settings primarily in HICs (though not specific to PLWH), as well as trauma-informed care (TIC) within RWC settings in the southeastern U.S.

Mutschler et al. (28) conducted a systematic review of the literature on the implementation of peer support in mental health services and reported best practices in alignment with the domains of CFIR, identifying potential barriers and facilitators, as well as key process factors to adoption and implementation. However, little is known about the feasibility of integrating such programs into HIV care settings in the U.S (29). Past studies of TIC conducted within the Department of Health and Human Services (DHHS) Region IV RWC settings in the southeastern U.S. applied CFIR as a framework for analysis and identified numerous inner and outer setting constructs that may be key to implementing trauma-informed interventions for PLWH/T (30–33).

The present qualitative study applied a similar theory-driven approach to assess the feasibility of implementing or expanding peer-delivered MHPSS interventions for PLWH/T in HIV care settings using the updated Consolidated Framework for Implementation Research (CFIR 2.0) by Damschroder et al. (34). In this paper, we report upon the barriers and facilitators identified from the analysis of key informant interviews conducted with administrators, providers, and staff at RWCs across the southeastern U.S.

## Materials and methods

This study was approved by the Emory University Institutional Review Board (IRB 00006376). All participants gave informed consent (written and verbal) prior to participation and were provided compensation for their time. The reporting of our methodological approach and findings was guided by the consolidated criteria for reporting qualitative research (COREQ) (35).

### Study design and context

This study is part of a larger mixed methods study assessing the acceptability and feasibility of implementing violence and PTSD screenings, as well as MHPSS interventions in RWCs and community-based organizations for PLWH/T. Qualitative key informant interviews, guided by CFIR, were conducted with a subset of RWC administrators, providers, and staff across the southeastern U.S. (i.e., DHHS Region IV: Alabama, Florida, Georgia, Kentucky, Mississippi, North Carolina, South Carolina, and Tennessee) who completed surveys as part of quantitative data collection.

#### Theoretical framework

CFIR is a multi-level, meta-theoretical framework that synthesizes common constructs across published implementation theories and provides an overarching typology of 39 constructs across five domains (i.e., intervention characteristics, outer setting, inner setting, characteristics of individuals, and process), allowing researchers to select constructs that are most salient to their study setting to guide diagnostic assessments of implementation context [Damschroder et al. (27)]. In 2022, CFIR 2.0 was published based on user feedback, and now contains 48 constructs and 19 subconstructs with some original constructs removed or revised and others added (34).

Although this study was conceptualized prior to the release of CFIR 2.0 (34), we were able to identify constructs corresponding with the original definitions in the updated version with a few exceptions (i.e., implementation climate, readiness for implementation, and leadership engagement), which we still included in our approach to data collection and analysis.

### Recruitment

Administrators (i.e., managers and decision-makers), mental health (e.g., psychiatrists, therapists, counselors, licensed clinical/medical social workers) and other clinical providers (e.g., physicians, nurse practitioners, physician assistants, certified nurse midwives), and staff members (e.g., nurses, social workers, case managers, medical assistants) were purposively recruited from among survey participants who indicated they'd be willing to participate in interviews (our sampling frame) to achieve diversity in roles across different states and organizations (including RWCs). One hundred forty survey respondents were contacted for interviews by email but 95 did not respond. Of the 45 interviews scheduled with participants from RWCs and community-based organizations, 44 were completed (including five pilot interviews), with one RWC-based interviewee experiencing technical difficulties early on (we were unable to re-establish a connection and reschedule the interview). For more information about survey and interview recruitment, including inclusion and exclusion criteria, see Garbarino et al. (36).^2^

Interview participants were contacted by email to schedule a two-hour appointment (the expected duration of the interview) and were sent a Zoom calendar invitation with instructions for accessing the virtual conference room. A reminder email was sent the day prior to the interview to confirm attendance.

### Data collection

Semi-structured in-depth interview guides were developed based on 31 CFIR constructs that were found to be salient in past studies (30–33) concerning the implementation of TIC in RWCs. Guides were first pilot tested with five participants (not included in our sample) and adjustments were made to improve length, flow, and clarity of questions based on participant feedback that was collected after each interview.

Interviews were conducted virtually over Zoom by two cis-female members of the study team with MPH degrees, **CWK** (also a PhD candidate) and **CKE**, between January 2, 2024-October 30, 2024. A master's-level public health graduate student was also present, serving as notetaker. Study team members were introduced to participants, whom having previously given written consent when participating in surveys, were read a description of the study, interview, and risks/benefits of participating before being asked for their verbal consent to participate and to be audio-recorded prior to the start of each interview.

The interview guide covered a variety of topics, including violence and PTSD screening, current MHPSS services, the acceptability and feasibility of implementing peer support interventions (i.e., peer mentorship/navigation and peer support groups), and the acceptability of peers as service providers for CBT and PFA/MHFA. For the purpose of this study, we conceptualized peers as PLWH that possess familiarity with accessing and utilizing MHPSS services through training and/or lived experience. Descriptions of each intervention (Supplementary File 1), training/certification cost and requirements, and findings about intervention acceptability among focus group participants were displayed and read to interview participants, who were then asked to respond to questions about the feasibility of implementing them at their clinics.

After interviews were completed, participants were offered $75 Visa or Mastercard e-gift cards in compensation for their time. Audio recordings were transcribed by a third-party professional service, Ubiqus. Transcripts were then cleaned and de-identified by master's-level public health graduate students for analysis.

Data collection continued until saturation was reached after completing 24 interviews with RWC participants and the decision was made by principal investigators (**JMS** and **ASK**) in consultation with **CWK** and **CKE** to halt interviews when no new information was forthcoming. No interviews were excluded based on audio nor transcription quality.

### Data analysis

Based on key constructs identified by past studies (28, 30–33), as well as a close reading of transcripts, a deductive codebook was created to assess 28 CFIR 2.0 constructs across its five domains, which were salient to the acceptability and feasibility of implementing peer-delivered MHPSS services: (1) innovation (i.e., innovation evidence base, complexity, relative advantage, adaptability, and cost), (2) outer setting (i.e., partnerships & connections, external pressure, local attitudes, policies & laws, and financing), (3) inner setting (i.e., structural characteristics, relational connections, communications, culture, recipient-centeredness, tension for change, compatibility, relative priority, available resources, and access to knowledge & information), (4) individuals (i.e., innovation leads, innovation deliverers, innovation recipients, need, and capability), and (5) implementation process (i.e., planning, engaging, and doing). Three constructs from the inner setting of the original CFIR (i.e., implementation climate, leadership engagement, and readiness for implementation) were also assessed. Codebook definitions were developed and tested on the pilot interview transcripts before being refined and finalized with instructions for coders; interview participants did not contribute to transcript review nor codebook development.

Transcripts were uploaded to MAXQDA 24 and independently coded by **CWK** and **SCG** (a PhD student with an MPH degree), both with training and experience in qualitative research, including CFIR studies. Intercoder agreement was established using a consensus approach with coders meeting to review and discuss transcripts. A third reviewer, **JMS** (PhD) or **ASK** (MD, MSc), was brought in to resolve any differences in coding and answer questions requiring subject matter expertise related to CFIR or clinical practice in RWC settings respectively.

For this study, we conducted qualitative thematic analysis on deductive codes related to feasibility of implementing or expanding peer-delivered MHPSS services using a CFIR 2.0 lens. Although these codes were primarily assigned to one question, they were more broadly applied where relevant. Specifically, we examined codes about feasibility, barriers, and facilitators for each peer support intervention (i.e., peer mentorship/navigation and peer support groups), as well as task shifting codes for CBT and PFA/MHFA (i.e., acceptability of lay health workers, such as peers, to deliver the intervention) and the code for which MHPSS intervention was most feasible to implement. Coded segments were paraphrased and compiled in a Microsoft Excel table by participant, which was used to summarize themes that emerged across participants and to identify the most salient CFIR constructs, which are described in the next section.

## Results

In total, 24 RWC health care workers (HCWs) participated in interviews, which averaged 110.5 min (range: 59–137). Most were staff members but mental health providers, clinical providers, and administrators were also present in our sample. Participants who chose to disclose their gender and race identified primarily as female and were equally Black or White. Interviewees were mostly based at health system/hospital-based HIV/ID clinics and standalone HIV/ID clinics or community clinics, although the sample also included HCWs at federally qualified health centers and health departments. All eight DHHS Region IV states were represented in our sample.

The most salient CFIR constructs as they relate to intervention feasibility, including implementation barriers and facilitators, for each peer-delivered MHPSS intervention are summarized and reported below (see Supplementary File 2 for construct definitions and operationalization). Additionally, we report findings concerning which MHPSS interventions are most feasible to implement in relation to peer support. Exemplar quotes are provided, along with participant ID number and role at clinic (i.e., A = administrator, MHP = mental health provider, CP = clinical provider, and S = staff member) for context; we did not observe notable differences in responses across roles.

### Peer mentorship/navigation

Most participants believed that implementing peer mentorship/navigation for the purpose of improving clients' mental health and providing them with psychosocial support was feasible (*n* = 16, 66.7%), though some conditioned it on funding, being able to find the right peer, peers having proper training, and needing to think through overcoming past challenges with implementation. A few participants (*n* = 3, 12.5%) needed additional information to determine whether implementation was feasible or not, and some participants (*n* = 5, 20.8%) felt that it would not be feasible due to the level of effort needed, funding challenges, limited space, limited clientele, and difficulty finding a peer mentor/navigator. Innovation complexity, the ease or difficulty of implementing or expanding the intervention, was an overarching theme. Additional barriers and facilitators are reported by CFIR 2.0 construct.

#### Engaging

Engaging and its subconstructs, *innovation deliverers* and *innovation recipients*, were key barriers to implementation. Those interviewed reported challenges finding people willing to serve as peer mentors/navigators and clients willing to receive this intervention, often in relation to the construct of *need*. However, several participants discussed approaches to engaging these groups, which could facilitate implementation of the intervention.

A couple of participants reported that their clinics were engaging clients through events and pop-ups where there are screening/testing services, offering them financial incentives/compensation to attend or opportunities to interact with peers who would be in attendance so that they could become known by the community/prospective innovation recipients. According to one mental health provider, better *communication* or more “word of mouth awareness of who we are would be helpful in facilitating this kind of program” (326-MHP).

Additionally, one staff member reported that clients have been informally pairing up with each other while at her clinic, and that it could be helpful to adapt the intervention to incorporate peer mentorship/navigation into peer support groups, where they can get to know each other through social activities:

…they just became buddies and then they became each other's support. Then they kind of became each other's peer mentors sort of, and they didn't even mean to but it all started with them having some fun together. (67-S)

#### Innovation deliverers

Participants discussed several barriers related to this subconstruct. Some participants reported difficulties with hiring peers due to turnover (e.g., re traumatization), external stigma (e.g., living in the “Bible Belt”), and internalized stigma (e.g., many clients aren't comfortable with nor accepting of their HIV diagnosis). As one administrator explained:

I think it's great on paper. *[Laughter]* But as far as my experience, I was trying to hire peers to work in this role. We have not been quite successful…the last peer that I hired, was–didn't last with us, but a few months. He thought that he was at a point to where he wanted to give back to other peers. But instead, it actually kind of triggered him to just talk about HIV every day, all day. So, it was actually the opposite effect…and then also, geographic location, you know, we're smack dab in the middle of the Bible Belt. Stigma is very, very huge, and a lot of patients are afraid to share their diagnoses or disclose with others for fear of intentional disclosure. (106-A)

Additionally, stigma impeded engaging peers because it could be challenging to find someone willing to disclose personal information about themselves with others, communication being an essential component of this intervention. Furthermore, a couple of mental health providers voiced concerns about the role of innovation deliverers, including “blurring the line” between being both a peer and employee (282-MHP) and innovation recipients “relying too much on the peer navigator” when disclosing information (330-MHP), though the participant did not share her team members’ concern. A few participants raised important questions related to peer selection and the hiring process that they perceived as obstacles, including whether they had candidates that were a good fit for the role in terms of lived experience (e.g., do they match the client demographics, such as age, gender/sexuality, race/ethnicity, location, or mental health condition?) and “…do they come from your patient population? How are you going to hire them? All that kind of stuff. You can't do it without answering those questions.” (67-S).

Participants spoke of the need to bring on more peers and to find the right person to fill the role: someone who is comfortable and at a place mentally and emotionally to share their story with other clients without being triggered, is accepting of their diagnosis, is relatable to clients (e.g., needing more peers who are Black/African American men who have sex with men), has the right personality, and is willing to become a mentor. A few participants were concerned about potential peer mentors/navigators being comfortable with the job description (e.g., having an HIV diagnosis and mentoring PLWH/T), which one participant mentioned was posted to the general public, and getting the training for it.

However, RWCs varied in their ability to engage peer mentors/navigators, and a few providers shared potential facilitators for engaging innovation deliverers. One clinic had many potential candidates to draw from within their client population. Another clinic had staff that recommended people for the role that they thought would do a good job (though the identified peer mentors/navigators were not very successful transitioning into the role). One clinical provider suggested engaging innovation deliverers from among innovation recipients:

I do think there are some barriers but I think it's a good place to start. Especially if perhaps, it could be like a time limited commitment or something, so you're not signing up to be a buddy for the next 10 years. But you're signing up for like, yes, I'm going to mentor you through this for six months or 12 months or whatever. Then maybe have the expectation that then the person who was mentored would go on to mentor someone else, that's like training the trainer. (192-CP)

#### Innovation recipients

Participants identified similar barriers to engaging innovation recipients, such as discomfort with disclosure (e.g., they had difficult things happen to them or they might prefer a professional), stigma, and potential for retraumatization. Additionally, privacy and safety were concerns that might prevent clients from accessing services, especially if they were experiencing violence at the same time:

Depending on their situation, I feel like people could potentially have more violence towards them, if the person perpetrating the violence found out about it. I think that privacy and disclosure could be a huge concern, especially if violence was the issue. (192-CP)

We have one patient in particular that comes to mind, who has trauma, previous trauma. And she's still currently with it. And we literally would have her come into the office and, you know, she expressed to us what's going on at home. But she will get on a public transportation bus and go right back to that residence. So, I don't see him allowing her to attend or to meet with a peer mentor. So, I think that would be a barrier, like the living arrangements, because sometimes we have those who are actively still in a violent situation. Or even in their community, and it's just hard to pull away when it's still occurring. (258-S)

One staff member suggested that taking a more comprehensive approach would facilitate client engagement:

I think educating each encounter with the client, letting them know that this is available, that you can talk to others, this is a safe space, we're going to help you as much as we can–providing assistance with that, as well as implementing group sessions with the peer advocate leading or having them enter in the encounters with the client and the case managers and the providers. Also having resources outside the waiting room so clients can sit and look through them, become aware of these resources. We have a pamphlet that details each service that we provide here at the [Organization 51]. So, I think that's a couple of ways to help. (379-S)

#### Need

RWC clients also might not engage in the intervention due to barriers, such as inability to access needed MHPSS services or their level or perception of need. Transportation was mentioned several times as a barrier to engagement (e.g., issues with the public transportation system and older clients needing transportation support because they prefer in-person to virtual engagement). Continuity of follow-up and services was perceived to be difficult because clients were sometimes transient and communication could be a challenge (e.g., phone numbers would get disconnected, preventing linkage to services). Additionally, some clients may not be ready to address their mental health or they may feel ok in the moment and not understand how the intervention will help/prevent them from experiencing a crisis later. They may lack the support (e.g., not having access to stable care or outside support) to seek help or feel that too many people are already involved (e.g., police or the Department of Family and Children's Services). Others may simply have other commitments and obligations that get in the way of prioritizing help-seeking.

However, a few participants thought that demonstration of need among the community they serve would be a facilitator of implementation or expansion. One administrator shared that her RWC conducted market research to see who they were servicing and that results from its needs assessment may determine whether the intervention would be expanded:

And we've more recently–we looked at who we're servicing–the population, in addition to what zip codes, and that has really worked with us being able to create locations in those zip codes. And then depending on the population that comes into those zip codes. Which 100 percent of, you know, all our locations have the HIV population. So the need for peer support will be based on, you know, the number of clients that we have increased and the number of clients that we're currently seeing. And if we need more. (314-A)

Another participant, a staff member, mentioned that while COVID-19 was previously a barrier, they now had more clients coming in with comorbidities (e.g., substance use and addiction) and needed to look at expanding their peer support program because things were reopening.

#### Recipient-centeredness

This construct was often discussed in relation with the construct, *engaging*, as a potential facilitator to implementation. Several participants thought that catering to client interests could be a motivating factor for clinics. A couple of participants wanted to assess acceptability of the intervention, with one mental health provider suggesting client surveys to see if they would like to have or be a peer mentor/navigator. Others believed, based on their knowledge of clients, that they would prefer peer-oriented services and want to participate.

Implementing recipient-centered practices was seen as a possible way to overcome client resistance to peer mentorship/navigation. A couple of staff members thought that clients may prefer different approaches to engagement, e.g., existing clients may need more convincing/education about intervention pros and cons/perks/tailoring than new ones and others may need to be allowed space to come to their own decision (i.e., continue to educate them but respect their choices because being too pushy could lead to resistance). Another staff member suggested warm handoffs to facilitate disclosure and linkage to care.

#### Financing

Financing was a key factor that could be both a major barrier and facilitator of implementation or expansion of peer mentorship/navigation. Some participants reporting limited financial resources (funding being a subconstruct of *available resources*) in relation to the construct of *innovation cost* (i.e., compensating innovation deliverers/leads and paying for client transportation). The need for funding/financing was often discussed in general terms, making it difficult to ascertain whether participants were referring to internal or external sources. However, several participants mentioned receiving external funding from Ryan White and grants from other sources, such as the Substance Abuse and Mental Health Services Administration and the National HIV/AIDS Strategy (NHAS).

Funding sources sometimes required activities to be in alignment “with what the grant calls for” (411-S) and new interventions and services to be written in to proposals. One clinical provider described how the rigidity of grant cycles could make it difficult to introduce changes and expand services, as well as how even with funding, they lacked control over how those funds were used:

…the thing with our clinic is everything's pretty much locked in based on grant cycles. And the grant cycles aren't always the same…you can't just necessarily add people in the middle of a grant cycle because…you're locked in for the period of the grant. And then if you want to try to expand it, then I guess you write a different kind of grant or ask for more money for next time, but in the middle of the program in the middle of a grant cycle, you have what you have, and you can't really get more…And a lot of our financials, like we don't work like the other clinics because we're Ryan White funded, we're grant funded. [Hospital 6] doesn't pay the salaries. Ryan White pays the salaries, but still the main hospital, the [Hospital 6] administration takes all the money that we get, that we make, and we don't keep it on site. So, we don't have as much autonomy as we used to make those kinds of decisions. And so, everything has to get run by the C suite executives downtown, like everything we do. So, even if we want something, we don't necessarily get to have it, even though we're providing our own funding. (313-CP)

Still, for most participants, existing grant funding or applications for funding were seen as facilitators of implementation and expansion of peer mentorship/navigation. Many participants reported that their clinics had the funding and ability to implement or expand peer mentorship/navigation. In addition to grant funding, one mental health provider mentioned that savings from his clinic's 340B pharmacy expansion (i.e., rather than pay 40% of their funding to another pharmacy, it stays in house to allow for job creation) would allow it to potentially hire between one to three peers. A few participants reported already being in the process of hiring peers or expanding, including one clinic that received a grant specifically geared towards expanding peer counseling and navigation.

#### Available resources

Having a “tight budget” (258-S) and needing money/funds were reported by some participants as a barrier but other RWCs were well-resourced. In addition to funding, participants described the availability of other clinic resources, which could pose as either a barrier or facilitator to implementation or expansion of the intervention. Although several clinics did not have a community advisory board (CAB) in place, many clinics did have one and/or quality assurance/control committees, where client and peer members could provide input and feedback from a patient perspective. One mental health provider mentioned that his clinic's CAB was also its Health Resources and Services Administration-required quality control group, and that implementation of the intervention would be discussed within this entity. Another mental health provider reported that her clinic's peer advocates served on the CAB and also recruited other clients that they thought would be good to have on the board. One staff member mentioned that they were in the process of reestablishing the CAB, which would be integral to the planning process, and of which peers used to take part.

Physical space was also discussed by some participants, and although a couple of clinics lacked space within their buildings and facilities, it was not a problem for others. Additional barriers included a shortage of time to supervise the intervention and for people to access these services (e.g., limited hours of operation for enrollment and continuity clinics), as well as a general lack of resources beyond Ryan White and what was available in the community to the public. Facilitators included offering free transportation to clients and resources from pharmaceutical representatives. However, one administrator noted that Ubers were expensive, so it was not a long-term solution, and that they also had multiple locations to improve access to services.

#### Structural characteristics

This construct was primarily discussed as a barrier to implementation or expansion. Several participants reported issues with staffing preventing them from expanding peer mentorship/navigation services at their clinics, describing a significant amount of burden placed on an inadequate number of peers, limiting their availability to serve clients.

…she is the only one there. Like we could have like two–a patient having a crisis, like two or three patients having a crisis, and she has to pick priority. Versus if we had several, you know, peer mentorships, then one of the peer [mentors] could have been with one patient and another [with the other]. (283-S)

Availability of the peer, I think. Just–we just have the one, and like I said, she can't be everywhere at once, and we have multiple–she serves clinics in multiple locations. So, we have three separate HIV clinics that happen on our campus, and she kind of serves all of them. So, just availability is probably the biggest barrier. (268-S)

Conversely, one mental health provider (330-MHP) mentioned that because they had a smaller client list due to limited grant funding, there was less of an interest in resuming peer mentorship/navigation services and hiring a peer.

Although many clinics were already implementing peer mentorship/navigation, several participants expressed the need or desire for having more peers on staff due to the level of demand. In a few cases, peers were serving multiple locations because there weren't enough to have one at each site. One mental health provider thought it would be important to conduct a needs assessment when considering service expansion:

I guess just determining, you know, with the current ones we have, what does their caseload look like? Do they feel they have enough time to give existing clients adequate attention? And if not, you know, do we need to bring someone else in? (282-MHP)

However, a few participants reported that their clinics had the infrastructure for peer mentorship/navigation (e.g., an established role, having a comprehensive framework and resources to support the position, being the largest HIV care provider not affiliated with a local hospital). One participant expressed a preference for the intervention to be implemented in a structured location (on-site) in case transportation was a barrier for clients.

#### High-level leaders and opinion leaders

Participants primarily discussed the need for buy-in as a potential barrier to implementation or expansion. Although a couple of participants reported that clinic administrators would be supportive of implementation, many felt that they would need buy-in from leadership and other stakeholders to implement the intervention due to how decisions are typically made in the clinic:

I would say probably funding and probably more support from upper leadership to kind of support that. Because in the clinic we can kind of say a lot of things about what we need, but at the end of the day it's from the top down. And so it's kind of like sometimes we always feel like we have a need and we have to work so hard to get a little bit. (2-S)

As one mental health provider noted, when administrators are involved, “something has gone wrong, I would say. There's some kind of conflict or issue between clients” (326-MHP). A staff member observed that in these cases, it may take reassurance that things will be different to get them to support the intervention again due to poor implementation in the past:

I think overall buy-in is going to be very important because we have run into some issues with our peer program historically, and many of our–not many–some of our staff and faculty, providers and such have been here for quite a long time. So they're aware of the issues that have come up in the past or what that role looked like in the past and may have expectations of it looking the same or they don't want it to look like that. So, because of our history with peer navigation, we would have to kind of navigate that. (67-S)

A few participants mentioned that providers needed to be consulted or be on board as relationships between peers and providers could be challenging, though they had the potential to be mutually beneficial and supportive. Additionally, a clinical provider felt it important that there was training and awareness about why they were doing it so that everyone understood “…why are we doing this? What will this do for patients?” (313-CP).

#### Access to knowledge & information

Participants discussed this construct as both a barrier and facilitator of implementation or expansion. Those interviewed often mentioned a need for training and supervision for peer mentors/navigators, which was appropriate for the job description, specific to domestic violence, and included assessment of peers' comfort level to “get them up to speed” (332-S). However, this could be burdensome to other HCWs due to lack of availability to train them or the need to improve their *capability* of doing so:

…we would need to train them. They would need some sort of supervision for sure. Which would mean staff being available to do that. I hadn't thought about those things. Those could be barriers…I just remember a conversation about hiring an LAPC and it was that we didn't have anyone supervise in the clinic. So busy, so I have a feeling it might be kind of–that it would be like that it would be like that if we were to have this peer program, [we would] need to have them supervised. (550-MHP)

…I don't know if anyone currently on staff has training in how to train peer counselors. I know I don't. I'm pretty sure that my supervisor doesn't, and some other folks might simply from prior experiences. (326-MHP)

One mental health provider also mentioned that implementing peer support trainings in hospital-based clinic settings could be hindered by *policies & laws*, such as the Health Insurance Portability and Accountability Act (HIPAA; i.e., patient privacy and confidentiality).

However, many participants reported that their clinics had the resources and were willing to train and/or supervise peer mentors/navigators if they weren't doing so already. A couple of participants mentioned that training could be easily resourced (e.g., a former peer would likely help). One staff member mentioned that there was a standardized state-wide training program for peers and another mentioned that the clinic's peer supervisor (a data analyst who started the program, though previously it was the clinical therapist) met monthly with peer mentors/navigators to check in. One administrator reported that their peers had group supervision once a week and individual supervision once a week or as needed. Additionally, a couple of participants discussed the importance of educating HCWs that peer mentorship/navigation is needed, as well as acknowledgement that the need exists.

Educating stakeholders was seen as important to establish “not just cost effectiveness but also how would having this additional resource for our clients improve the quality of service that we could provide our clients? Like I don't think it could, sadly enough, I don't think it could exclusively be what would this do for our clients, but how will this help us as an agency?” (326-MHP). Understanding the value of peer mentorship/navigation was crucial to getting buy-in:

Training and demonstration of, like, why are we doing this? What will this do for patients? You have to get the buy in. Because you can't just slap another program on people. We get a lot of that. We get a lot of things sort of like oh, and now we're doing this. I think things work better if everybody understands why and you have the buy-in from the people who are administering the program. We are doing this because we really want to improve our patients' experience. We want to improve their overall health. We want to improve their mental health and allow them to be successful and be healthy or whatever it is you want to say. But there needs to be more of that because when you just throw more work on people without really explaining it, you don't always get great buy-in. (313-CP)

One clinical provider wanted an expert to visit and show people how easy it is and that maybe they are already doing it informally–someone who has a good foundation and can build confidence.

#### Partnerships & connections

This construct was discussed by participants as a facilitator to implementation. One mental health provider thought that if [Organization 52] had a peer support program, it would eliminate some issues her clinic faced from being hospital-based. Another reported that her clinic stays in touch with different nonprofits locally, as well as with the clinic's former peer navigator, and that they would be well connected to find a qualified peer if they wanted but must maintain a good relationship with community resources to do so. One staff member mentioned that her clinic has connections with other RWCs that provide the intervention and would be good resources.

### Peer support groups

Participants had mixed perspectives on whether peer support groups could be feasibly implemented or expanded at their clinics. Nine participants (37.5%) thought that the intervention would be feasible and six (25%) thought it would not be feasible. The remaining participants (*n* = 9, 37.5%) were uncertain, including one participant whose clinic was already implementing the intervention to good outcomes, though they were having difficulty justifying their work and did not think it feasible to expand. Participants identified several barriers and facilitators across CFIR 2.0 domains, the most salient of which are reported by construct.

#### Engaging

As with peer mentorship/navigation, engaging innovation recipients was an important barrier and overarching theme for peer support groups, including its subconstructs, *innovation recipients* and *innovation deliverers*. Related CFIR constructs were *need* and *recipient-centeredness* in relation to recipients and deliverers respectively and are reported together with these subconstructs.

Maintaining sufficient group membership and ensuring consistent attendance were seen as challenging for RWCs and were crucial to successful implementation, as small group size could be discouraging for people who did show up. Participants felt that it would be good for peer support groups to be ongoing rather than term-limited so that clients could join as needed. One staff member suggested setting a meeting date so that transportation could be arranged for clients in advance.

#### Innovation recipients/need

These constructs were often discussed as barriers to intervention engagement among clients. These barriers included comfort level, stigma, privacy and confidentiality, safety, and trust, which were often intertwined. Stigma could prevent people from coming to groups if they don't want to be seen entering a building associated with PLWH and the LGBTQ+ community or if they have trauma histories and are afraid to be in public or acknowledge they have experienced violence. The ability of RWCs to address safety issues and ensure confidentiality were mentioned by some participants as potential facilitators to engaging innovation recipients, as clients needed to feel like they could trust the clinic to heed their concerns, especially if the clinic was well known within the community.

However, some populations were more difficult to engage. One mental health provider noted the difficulties engaging people who have been exposed to or experienced trauma:

…you know, people with trauma, they are going to avoid. That's the main symptom. Any type of–main trauma diagnosis, avoidance, they don't want to talk about it. They're trying to manage their emotions, their mood. They're trying to manage the best way they can. And as long as they're in control and things are okay, they don't want to talk about it. They're not going to engage in certain services. (214-MHP)

The same mental health provider also noted that they struggled to generate enough interest in peer support groups among transgender clients and among young clients who did not care for the group format. One staff member also experienced challenges implementing an alcohol and drug group because “people wouldn't come because it was so specific” (213-S). A couple of participants thought that client knowledge of the intervention and access to services were obstacles, with one staff member noting difficulties with “…just getting them there, getting them to believe that this is important and helpful” (359-S). One clinical provider also shared that surges in COVID-19/flu were a deterrent, as people “aren't totally jonesing to come together that much” (91-CP).

Another key barrier to engagement was the ability to access needed mental health support. Clients struggled with transportation and attending meetings in-person, especially if they had to travel long distances or lived in rural areas. Some clients, such as younger people, don't drive or lack transportation, and one mental health provider complained that “[City 8] has possibly the worst public transportation of any large metro city I've ever lived in” (326-MHP). A couple of participants thought that meeting on a weekly basis would be difficult with these transportation challenges.

Finding a time to meet was also a barrier for a few clinics due to scheduling challenges. Meetings during working hours resulted in lower engagement among clients. Additionally, due to how clinic services were structured, clients weren't seen regularly, making it difficult to advertise services or hold regular meetings. Time was also in limited supply for both innovation recipients and deliverers. If people were too busy or unwilling to come, then clinics couldn't justify providing it because it wouldn't be a good use of their time.

Client interest in the intervention and attendance were also discussed by some participants as potential facilitators for implementation at their clinics. One staff member mentioned that their clinic had the community for it and some interest. If there was a need or demand for it (clients asking for/requesting these services), then clinics would consider implementing or expanding the intervention, if feasible to do so (e.g., capacity and enough people to participate). Another staff member thought that clients may need time to realize that the intervention was good for them to seek out the intervention. To improve participant attendance, one mental health provider recommended having a peer support group leader be someone that clients were comfortable with and a clinical provider suggested that groups meet less frequently, proposing monthly or quarterly meetings instead.

#### Innovation deliverers/recipient-centeredness

Engaging innovation deliverers was discussed more often as a barrier to implementation with recipient-centeredness as a facilitator. Participants mentioned they had difficulty finding clients that fit the criteria to be peer support group leaders (e.g., past but well-managed trauma, relatable/representative of the client demographic, willing to take the time to do it, having training/be willing to complete training), with one administrator explaining that they may prefer to join the group as members. Another administrator emphasized the need for a peer leader who could build rapport with other clients because they may be slow to engage or might not engage if they don't trust the staff or the group.

A few staff members mentioned that they needed more staff availability. One explained that though they had a peer, he didn't have the bandwidth to also facilitate a support group on top of working a part-time job, trying to find housing, and providing medical accompaniment to clients. Another anticipated institutional challenges with hiring related to compensation of peers, and a third was unsure of who would be able to facilitate if neither she nor the doctor were present.

Participants were primarily concerned with finding suitable peer support group leaders and a couple felt that once they were identified, everything else would fall into place and they would be ready to implement soon after. Participants identified several recipient-oriented criteria they would like to see in a peer support group leader, such as being someone that the clients were comfortable with, the ability to build rapport with the client (including engaging with people individually before going into the group setting if needed), lived experience (e.g., be relatable and have past trauma that is well-managed), a desire to help other people, and an interest in, commitment to, and willingness to do it.

Several participants emphasized the importance for participants to either have training or be willing to take the time to be trained. A couple of participants thought it was important that peer support group leaders were “comfortable with referring back” (332-S) and had good facilitation skills: “knows how to redirect, how to bring people together if they–if people start to get kind of triggered” (91-CP). One mental health provider mentioned that her clinic would be able to get recruitment assistance from an affiliated organization of their institution (its outreach arm), which could help in identifying people within the community that don't qualify for RWC services but have the lived experience.

Participants felt that clinics and the administration would support implementation if they felt it was good for client care because they “want to provide” (304-MHP) for clients and “they're open to, you know, sort of any idea that somebody comes forward with if they think it's going to help the community” (359-S). One staff member thought that clients would prefer peer-oriented services, which could facilitate implementation of peer support interventions.

#### Structural characteristics

Participants reported several structural barriers to implementation across its subconstructs, *physical*, *information technology*, and *work infrastructure*. With regard to *physical infrastructure*, participants mentioned challenges with location and space, such as access issues (e.g., inconvenient location and the building being closed after hours and on weekends), not being a standalone clinic (e.g., connected to a hospital and must follow their rules), and unavailable or uncomfortable meeting places due to lack of privacy or as one staff member shared:

We're in downtown in this medical hospital area. Our offices and our clinic [are] in the bottom of a parking deck. There's conference rooms, but then you have to park here and go into the bowels of the hospital. Ideally, we'd have our own standalone clinic with a lovely conference room with vending machines and stuff. (67-S)

Additionally, clinic size was mentioned as a challenge, as there were not enough clients to form a group and the clinic would need to partner with others to have the numbers. A couple of other clinics served wide areas or multiple locations, which made organizing meetings difficult.

However, several participants discussed this construct as a potential facilitator to implementation. A couple of participants shared that having a bigger client population would enable them to implement the intervention. One staff member thought it would be helpful if the intervention had more structure around it, and one mental health provider mentioned that his clinic was willing to invest in infrastructure for it.

*Information technology infrastructure* was also perceived as a barrier for engaging clients through virtual meetings due to lack of reliable internet access, perceived complexity of implementing virtual peer support groups, and lack of support for telehealth. Although one clinic tried distributing iPads to clients with a partner organization, “it didn't end well for some reason and we stopped that program” (282-MHP).

However, some clients thought that virtual support groups would facilitate engagement among clients due to privacy (e.g., keeping the camera off) and ability to participate from anywhere, though one participant thought it would be less optimal but still an option. A few participants shared approaches their clinics were taking to facilitate virtual support groups, such expanding access to and use of technology and online resources, e.g., paying for HIPAA-compliant Zoom accounts, enrolling participants in federal programs providing lifeline phone programs and internet service, or telling clients about apps that they can download through their phones’ store to access other forms of peer support groups. One staff member mentioned that their clinic could use the internet to handle overflow if facilities were at capacity.

Finally, *work infrastructure* was primarily discussed as both a barrier and facilitator to implementation or expansion in terms of staffing. Participants did not think there were enough peer support group leaders available to facilitate groups or hold them frequently enough without hiring additional people, expressing concern that peers were already spread too thin with other duties and that they were losing possible service providers to other clinics. Additionally, staff may not be available to supervise, which could pose a barrier to implementation.

Several participants thought increasing the number of peer support group leaders would enable them to rotate and share the burden, and a couple of participants mentioned that they have the people in place, including peer advocate positions and case managers to help with implementation. One mental health professional thought it would be helpful to have people licensed in mental health care to assist peer leaders and co-facilitate.

#### Innovation cost

Participants mentioned several expenses related to innovation implementation that could be a barrier for some clinics but were more often seen as facilitators, including paying the salaries or stipends of peer support group leaders, as well as providing transportation assistance (e.g., public transportation cards and Uber cards), refreshments (e.g., snacks, meals, UberEats food delivery), and other incentives (e.g., gift baskets and gift cards) to encourage attendance. One administrator mentioned that her clinic would not be allowed to provide cash like other organizations implementing peer support groups, suggesting grocery cards as a substitute, and a staff member also mentioned money for food would help.

A few participants mentioned that not a lot of expense was involved in implementation, with one staff member mentioning that support groups were basically free to provide in the past and another mentioning not having to pay for training. However, one mental health provider mentioned that her clinic tried to start a voluntary group started but no one wanted to lead it, reinforcing a potential need to compensate innovation deliverers.

#### Financing

This construct was discussed primarily as a facilitator to implementation and expansion. Several participants mentioned that they had outside funding from sources like the NHAS, the Ending the HIV Epidemic Initiative (a key component of NHAS), the 340B Drug Pricing Program, Ryan White, the local government (e.g., county), and other grants that could allow them to implement the intervention and/or train peer support group leaders. Others that didn't already have funding emphasized the need for it.

#### Available resources

This construct was discussed both as a potential barrier and facilitator to implementation, integration, and expansion of services. Although some participants came from well-resourced clinics, others lacked the funding and space to meet and conduct trainings. One participant mentioned that they lacked a CAB to help implement the intervention but a couple of participants mentioned that they used their CABs to get advice from clients and peers about what they need from the clinic and thought that this forum could be used to discuss implementation of peer support groups.

#### Access to knowledge & information

This construct was discussed primarily as a facilitator of implementation. Participants often spoke of the need for the training and supervision of peer support group leaders to ensure that the intervention was well-run, though one participant mentioned wanting a good leader, whether or not they'd been trained. Several participants shared that they were providing training already. One of the clinicians thought it would be pretty inexpensive to train clients because they already had the lived experience and one of the clinicians could train the facilitator to feel confident in their ability to support clients. In terms of supervision, one participant mentioned the need to have someone to report to/process things with, such as a medical case manager's manager. One administrator shared that the clinic's peers received comprehensive supervision from the clinical team:

So they have weekly clinical supervision with our clinical team so we can staff any cases and [see] how the group is progressing, and then they can have individual time with their supervisor to, you know, review any questions, concerns and then go over any professional goals. (314-A)

A couple of participants mentioned that they had people on staff who were familiar with how to set up and facilitate groups and could contribute to intervention implementation and oversight. One participant mentioned that they also had online resources that could help. However, several participants mentioned that they lacked sufficient training for peer support group leaders and a couple of participants mentioned a lack of supervision. Awareness among providers about the availability of this intervention was also mentioned as a potential barrier.

#### Communications

Several participants discussed the need for better marketing of the intervention, which could be either a barrier or a facilitator for engagement. One mental health provider mentioned that her clinic can disseminate information and reminders about the intervention and group availability (e.g., create flyers, talk about it, send emails), as well as let staff know to be on the lookout if they have a client that would be a good facilitator or member. However, one staff member mentioned that her clinic would need someone to help email the peers and coordinate the intervention. Another staff member thought it important that the intervention was advertised across not only across the division but also the institution, which could lead to more support and funding for the intervention.

#### High-level leaders

Receptivity to the intervention among high-level leaders was perceived by participants as either a barrier or facilitator to implementation. Many participants thought that their clinic administration/leadership would be very supportive of peer support programs, such as peer support groups, for reasons including familiarity with the intervention, its effectiveness, wanting to help the clients and the community, and alignment with mission. However, a few participants thought that they would need to get institutional support or board approval based on things like level of need or goals of care.

#### Innovation complexity

Although this construct encompassed most of the previously discussed barriers and facilitators, with regard to the establishment of peer support groups, it was discussed primarily by participants as a barrier to implementation. Although several participants didn't think there would be barriers to implementing this intervention, others mentioned that getting groups set up and maintaining them would be a challenge. Especially among participants that had never done it before, logistics and operationalization would be tricky and they would need to consider things such as: meeting time, meeting place, who will facilitate and how will they be trained, what topics will be discussed, and what happens when where is a crisis needing a professional. One clinic provider mentioned that her clinic was not currently screening for violence so it would need to start with that. A staff member mentioned needing to go through the process of getting approval from the board, which would require a lot of steps and would be challenging.

### Perceived feasibility of other peer-delivered MHPSS interventions

Although participants were only asked about the acceptability of CBT and PFA/MHFA delivered by lay health workers (e.g., community health workers and peers), several CFIR constructs relating to feasibility were described as potential barriers and facilitators and are reported by intervention.

#### CBT

Only five participants (20.8%) discussed the feasibility of mobilizing peers to deliver CBT. A couple of participants mentioned that peers, as *innovation deliverers*, could fill in for other CBT providers or be an extension of CBT services. Although one participant expressed some concern about the *capability* of peers to provide therapeutic services, a couple of participants noted that peers were already providing emotional support to clients and that they spend the most time/have the strongest relationships with them.

*Innovation adaptability* was also discussed, including incorporating CBT into *available resources*, e.g., peer support groups or as part of a CAB with peer leadership that facilitates some CBT groups. One participant mentioned that their clinic was *planning* to have peer educators on staff, though another participant was uncertain of what the intervention would look like (e.g., *communications* and *access to knowledge & information*) and how their clinic would proceed:

Again, it becomes, “Are they documenting that?” I guess it would have to be a peer support person that was highly trained. I think anyone can get the training and sort of know how to do it, but then it's like where does it go from there? (304-MHP)

#### PFA/MHFA

A third of participants (*n* = 8) discussed the feasibility of peers as *innovation deliverers* for PFA/MHFA. Nearly all thought that peers would be a suitable–or even preferred–provider of these services as a potential facilitator of *engaging innovation recipients*. However, a couple of participants mentioned barriers to having peers deliver the intervention, such as HIPAA (i.e., *policies & laws*) and *financin*g (i.e., peer funding being tied to a certain grant and needing to write a new one specifically for this purpose).

When discussing the *capability* of peers, one mental health provider appreciated that the intervention allowed non-mental health professionals to assist clients if they become stressed while at the clinic, although one staff member conditioned that peers should be involved only when things were “not really heavy” (332-S). A couple of participants mentioned that peers were already *doing* some of the intervention elements at their clinics already, such as initiating referrals, and that the intervention was well-suited to be piloted with them.

Participants also noted several *structural characteristics* that could act as facilitators of the intervention. A couple of participants thought that it would be helpful to have a designated position for peers to deliver the intervention as their only responsibility so that they would have the time and space to do it and do it well. Peers were preferred for this role because they would have more availability than other HCWs to attend to immediate needs or crisis situations and it would be better if they had 1–2 people available that were trained and comfortable with stepping in outside of the case manager and therapist to help “bear the load” (313-CP). One clinical provider also thought that these services could easily be integrated into enrollment at their clinic, where peer mentors/navigators are more present.

Additionally, several participants mentioned training (i.e., *access to knowledge & information*) as a facilitator, including one staff member who described situations where peers were allowed to be in the room with the therapist or case manager “…so they can kind of hear what's going on. And it kind of gets them prepped for certain things” (332-S).

### Perceived feasibility of peers as service providers

When asked which of the studied interventions they thought would be most feasible, two thirds (*n* = 16) of participants mentioned peer support interventions as being feasible or the most feasible to implement, including one clinical provider who thought that peer-delivered PFA/MHFA would be the most feasible option. Rationale for choosing these interventions included the desire to cater to *innovation recipient* preferences for *engagement*, little to no *innovation cost* and less expensive in comparison to other interventions, *available resources* (e.g., funding and space), and *innovation complexity* in that it would be easier to implement because they were already *doing* it or because it would require less administrative effort “being in their hands entirely” (213-S).

Notably, one mental health provider thought that peer support interventions would be the best choices for PLWH/T and that giving clients what they want could lead to better outcomes:

I actually prefer the idea of the peer support groups and/or peer mentorship/navigation. I like those two the best. I feel like there's been a need and we'll be providing a need. And then with the incorporation of incorporating the domestic violence or the violence and the trauma piece, I think those will be good vehicles for that…I think that patients have kind of expressed a desire for peer support groups. So I want to give the patient what they need, what they want. I think that it's all good and well for me to say cognitive behavioral therapy is great, it's science backed. But at the end of the day, I want to give the patient what they're expressing a desire for, because I think it's going to be more successful if the patients have a say in what services we are able to provide for them. (550-MHP)

## Discussion

**Figure 1:**
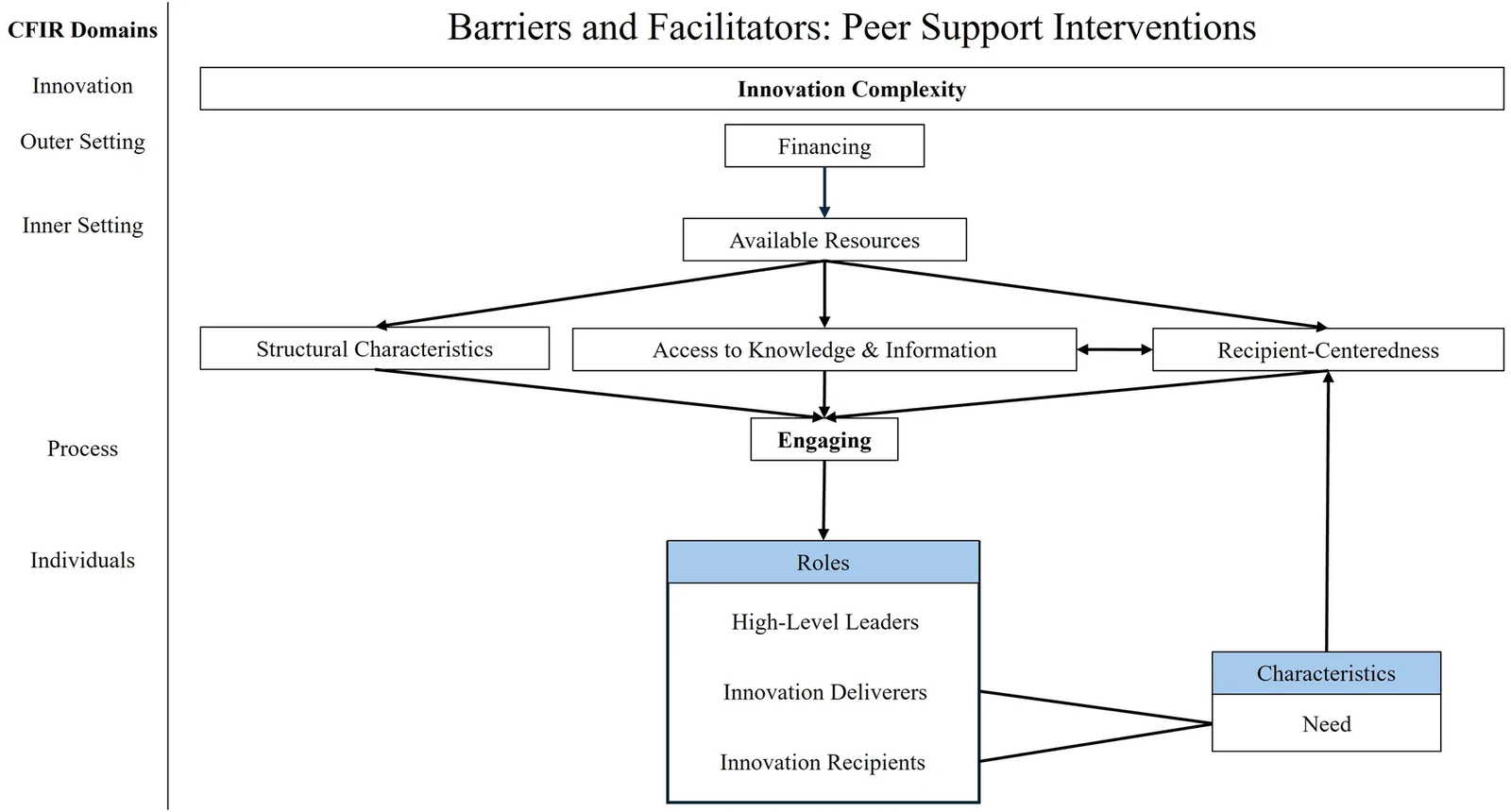
Conceptual model of barriers and facilitators to implementing peer support interventions.

Having peers as MHPSS service providers was considered feasible by the majority of key informant interview participants. We identified eleven constructs across the five domains of CFIR 2.0 (34), which could be barriers and/or facilitators to implementing peer support interventions (i.e., peer mentorship/navigation and peer support groups). A conceptual model of how these factors were related is shown in Figure 1.

The innovation domain construct, *innovation complexity* encapsulated both barriers and facilitators of implementation or expansion of peer support interventions within RWCs. In the outer setting, level of *financing* influenced the inner setting of RWCs by means of *available resources* (e.g., CABs, *funding*, *space*), which served as a determining factor for *structural characteristics*, notably its subconstruct, *work infrastructure* (e.g., the ability to engage peers as service providers and the availability of staff to supervise them), as well as clinics' ability to provide training (i.e., *access to knowledge & information*) and recipient-centered care (e.g., transportation and incentives to attend peer support groups) to engage *innovation deliverers* and *innovation recipients*, while accounting for their *need* (characteristics), as well as *high-level leaders* who have decision-making power over implementation and expansion.

Although the feasibility of peer-delivered CBT and PFA/MHFA were not assessed to the same extent, we observed that when participants spoke of barriers and facilitators, their perspectives further support observations made about peer support interventions, regarding engagement of innovation deliverers and recipients, as well as most inner and outer setting constructs.

These findings suggest that peer-delivered MHPSS interventions for PLWH/T are feasible to implement/expand at RWCs in the southeastern U.S. and reinforce findings from previous TIC implementation studies (30–33), advancing understanding of multi-level factors across CFIR domains known to influence the implementation of trauma-informed services in this setting. Additionally, we found that many of the key factors identified in the systematic review of mental health-related peer support by Mustchler et al. (28) were also relevant for RWCs considering implementing or expanding peer-delivered MHPSS services for this population. Notably, the constructs of financing, available resources, access to knowledge & information, and innovation deliverers were salient to both peer mentorship/navigation and peer support groups, and although CFIR 2.0 (34) no longer includes implementation climate and leadership engagement as constructs, we found that obtaining buy-in and support from high-level leaders was important to the implementation or expansion of these interventions.

RWCs should consider addressing barriers identified in the study or adopting practices that were considered facilitators of implementation/expansion, such as applying for funding opportunities that enable development and training (e.g., privacy and confidentiality) of a client-oriented and professionalized peer workforce, as well as provision of resources (e.g., transportation and virtual support options) and incentives to promote client engagement.

### Strengths and limitations

This study contributes to the literature on behavioral interventions for trauma and trauma-informed MHPSS interventions for PLWH, addressing a longstanding gap identified by LeGrand et al. (1) and Kokubun et al. (26) respectively. To our knowledge, it is the first study to assess the feasibility of several peer-delivered MHPSS services for PLWH/T in the U.S, which enabled the study team to better understand RWC HCW perspectives on multiple interventions. Additionally, all eight states within DHHS Region IV are represented within our sample, allowing for a comprehensive understanding of regional barriers and facilitators, although our findings are not representative of all states nor regions served by RWCs. Although the original CFIR framework (27) contributed to the development of the key informant interview guide and codebook, we applied CFIR 2.0 (34) to the presentation of our findings, thus promoting the advancement of implementation theory within this context and setting.

A limitation of our study was that we did not assess CBT and PFA/MHFA as comprehensively as peer mentorship/navigation and peer support groups, and that the question we asked about the mobilization of peers to deliver these interventions had more to do with acceptability rather than feasibility. Further research is needed to examine the feasibility of adapting these interventions to be peer-oriented, as focus groups conducted with PLWH/T indicate that clients were interested in receiving these services from lay health workers, including peers (11). The practice of mobilizing lay health workers to deliver CBT has precedent in LMICs (23, 37), and the perception of its feasibility in the U.S. should be assessed in more detail among key stakeholders (and expanded beyond HCWs to include policymakers, legal/compliance/ethics officers, and representatives of professional associations and health system governing/supervisory bodies) with regard to the establishment of training requirements for peers (e.g., competencies, evaluation, licencing, certification) and scope of practice (i.e., responsibilities/boundaries, legal liability, and ethical guidelines concerning risk to clients).

Additionally, although this study assessed the feasibility of mobilizing peers to deliver MHPSS interventions, we did not purposively recruit peer providers as interview participants out of concern for selection bias (i.e., overrepresentation within our sample of HCW roles within RWC settings and increased likelihood of a more favorable response to peer interventions). Peer providers were re-classified among survey respondents willing to be contacted for interview as staff members and recruited thusly; therefore, our findings should not be considered as representative of peer provider sentiments.

Another potential limitation of our study was social desirability bias. In a few cases, participants joined Zoom meetings from their places of work, which could have impacted their reporting if they felt pressure to describe their clinic operations more favorably. However, participants appeared to be very forthcoming and candid in their responses regardless of their role at the clinic, detailing the challenges and limitations they faced at their sites. The study team also provided assurances that interview participants would remain anonymous to encourage them to provide honest feedback about the interventions' feasibility.

### Future directions

RWCs remain critical safety nets for care of vulnerable populations, and ensuring access to and utilization of MHPSS services, as well as continuity of HIV care, is essential to the physical and mental well-being of the clients they serve. Task shifting and sharing interventions, such as peer-delivered MHPSS services are often grassroots, community-driven efforts born out of limited resources and a tension for change. While mobilizing peers may require some expenditure, these interventions merit further study, particularly related to cost-effectiveness (e.g., compared to traditional service providers), which is always a salient factor impacting RWC decisions to implement or expand services. Additionally, the billing of these services and pathways for the mobilization of a peer workforce for MHPSS service delivery should also be explored across payors (i.e., Medicaid, Medicare, and private insurance) within RWC settings, as further investment in a peer workforce may be needed to overcome barriers (i.e., licensing and certification). However, nonclinical peer activities are less likely than others (e.g., CBT) to pose substantial challenges for integration into payment systems.

Some participants expressed a desire to learn how peer mentorship/navigation and peer support groups were being implemented at other clinics, and we hope to share findings from our assessment of how these interventions are currently being implemented at RWCs, as well as RWC HCW preferences for implementation in a future publication.

### Conclusion

Through interviews with RWC HCWs, we were able to identify key factors that may influence implementation or expansion of peer-delivered MHPSS interventions. Overall, peer-delivered MHPSS interventions were well received and perceived as feasible to implement/expand by RWC administrators, providers, and staff. RWCs should consider adopting these interventions to provide client-oriented care to PLWH/T, as they are considered relatively low-cost (i.e., they can be implemented on a voluntary basis, grant-funded, and adapted based on resource limitations, though the professionalization of peers as service providers would require some cost consideration for compensation) and feasible to implement. Implementing and expanding peer-delivered MHPSS services could also help close the treatment gap caused by provider shortages and address the unmet mental health care needs of this population.

## Data Availability

Audio recordings and transcriptions are not readily available due to privacy concerns. Requests for data access should be directed to Jessica M. Sales (jmcderm@emory.edu).

## Footnotes

1 Kokubun CW, Ellison CK, Guardado-Menjivar V, Druss BG, Woods-Jaeger BA, Kalokhe AS, et al. Acceptability of peer-delivered mental health and psychosocial support interventions among people living with HIV with exposure to violencerelated trauma in Metro Atlanta: Findings from focus groups. (Unpublished observations).

2 Garbarino SC, Carlson MS, Kalokhe AS, Haardörfer R, Sales JM. Validating the capability to screen for intimate partner violence measure. (Unpublished observations).
