# Supplementary material for: Perceived feasibility of peer-delivered mental health and psychosocial support interventions for people living with HIV/AIDS at Ryan White-funded clinics in the southeastern United States: a Consolidated Framework for Implementation Research study of health care worker perspectives
Source: Front Health Serv. 2026 Aug 11;6:1896794. doi: 10.3389/frhs.2026.1896794 (PMC13503366; doi:10.3389/frhs.2026.1896794)
Supplement: Supplementary file 1 [file Supplementaryfile1.docx]

**Supplementary File 1: Description of MHPSS interventions**

| **Intervention** | **Description** |
| --- | --- |
| Peer mentorship/navigation | Peers often serve in a peer navigator or mentor role, helping other PLWH access mental health and other support services by sharing information, going with them to medical appointments, providing one-on-one social or emotional support, and giving practical advice as needed. These “buddy” programs provide more focused attention and tailored assistance to individuals, and these supportive relationships can benefit both the giver and receiver of support. For example, a client who experienced violence, such as a hate crime, may be paired with a peer mentor/navigator with understanding of their unique challenges to help them get the resources and support they need. |
| Peer support groups | Peer support groups can be a source of encouragement and assistance to PLWH and strengthen social support networks within the positive living community (PLWH that strive to maintain healthy behaviors and lifestyles to prolong quality of life). Under the supervision of a trained peer leader, groups meet on a regular basis to share their lived experience of living with HIV or mental health challenges and discuss personal struggles in a judgement-free zone. For example, a health care worker might refer a client experiencing post-traumatic stress after experiencing violence, such as gang violence or war/combat, to a PTSD support group to benefit from other people’s lived experience. Support groups are an important resource, providing comfort and constructive feedback, and participants can learn from a variety of perspectives and experiences. |
| Cognitive behavioral therapy (CBT) | CBT is a form of talk therapy where clients work with a mental health professional to discuss thoughts, feelings, and emotions during sessions to find solutions to improve how they respond and feel during difficult times. For example, clients can go to a licensed CBT counselor to talk about their experience with verbal, emotional, or physical abuse and learn about ways to cope and work through that trauma in their day to day lives. CBT has been successful in improving symptoms of depression, anxiety, and PTSD in PLWH. CBT can be done one-on-one (client and counselor) or in group settings with other PLWH. During sessions, individuals may learn about their mental health condition and develop skills such as relaxation, coping, stress management, and confidence. The length of CBT depends on what is right for the individual or group and may range between 5-20 sessions or as needed. While confidentiality is to be expected during individual or group sessions, in rare cases where clients pose a safety threat to themselves or others, the therapist may be legally obligated to alert authorities. |
| Psychological First Aid (PFA)/  Mental Health First Aid (MHFA) | Psychological First Aid (PFA) and Mental Health First Aid (MHFA) are interventions intended to provide initial help to people who are in crisis or distress, whether they have experienced a traumatic event or are just having a really bad day. Individuals trained in PFA or MHFA can provide comfort and stability to clients in the moment, listen with compassion and without judgment, inquire about immediate needs or concerns, provide education about coping mechanisms and available resources for mental health and psychosocial support, as well as practical help accessing these services. It is important to note that after initial contact is made, continued participation in this intervention is entirely voluntary. This form of crisis intervention is not a substitute for counseling, and participants are encouraged to seek professional help. Although, this intervention is brief, it offers connection to short- and long-term support and resources, including mental health, housing, legal, and other violence-related support services.    This type of intervention is designed for non-mental health professionals and can be administered by anyone who has received the appropriate training, including providers, staff members, and lay health workers (i.e., community health workers or peers) at the clinic/your organization. For example, peers can be trained in PFA /MHFA to provide compassionate care to clients that disclose they’ve experienced violence, such as domestic violence during a clinic visit. |
