# Supplementary material for: Perceived feasibility of peer-delivered mental health and psychosocial support interventions for people living with HIV/AIDS at Ryan White-funded clinics in the southeastern United States: a Consolidated Framework for Implementation Research study of health care worker perspectives
Source: Front Health Serv. 2026 Aug 11;6:1896794. doi: 10.3389/frhs.2026.1896794 (PMC13503366; doi:10.3389/frhs.2026.1896794)
Supplement: Supplementary file 2 [file Supplementaryfile2.docx]

**Supplementary File 2: Operationalization of CFIR 2.0 constructs**

| **CFIR Domain** | **Construct/Subconstruct** | **Definition*** | **Operationalization**** |
| --- | --- | --- | --- |
| Innovation | Innovation adaptability | How the innovation can be modified, tailored, or refined to fit local context or needs | Degree to which the intervention can be adapted (e.g., alterations/changes) to meet RWC needs and facilitate implementation or expansion (e.g., telehealth) |
|  | Innovation complexity | How complicated the innovation is (e.g., scope and/or nature, as well as number of connections and steps) | Perceived difficulty of implementing the intervention innovation (e.g., too many components/elements, administrative challenges, burden on RWC providers and staff) |
|  | Innovation cost | How affordable the innovation is (e.g., operating costs) | Costs related to the intervention and its implementation/expansion at RWCs |
| Outer setting | Partnerships & connections | How networked the inner setting is with external entities (e.g., referral networks, academic affiliations, professional organization networks) | The extent to which the RWC is networked with other organizations (e.g., external referrals and collaborations), as well as the nature of working relationships related to the delivery of MHPSS services |
|  | Policies & laws | Legislation, regulations, professional group guidelines and recommendations, or accreditation standards that support implementation and/or delivery of the innovation | External strategies to spread interventions, including the influence of institutional (e.g., hospital or university) and governmental politics (local, state, and national), policies, and laws (e.g., Ryan White and HIPAA) on RWC operations, funding, or support |
|  | Financing | Funding to implement and/or deliver the innovation from external entities (e.g., grants and reimbursement) | The availability of funding from external entities (e.g., Ryan White and other grants) to implement or expand the intervention |
| Inner setting | Structural characteristics | How infrastructure components support the functional performance of the inner setting. | The social architecture, age, maturity, and size of the RWC, as well as its capacity related to human resources or physical space  Note: Some aspects of physical infrastructure were captured within the operationalization of available resources and some aspects of information technology infrastructure were captured within the operationalization of innovation adaptability |
|  | 1) Physical infrastructure | The layout and configuration of space and other tangible material features |  |
|  | 1) Information technology infrastructure | The technological systems for tele-communication, electronic documentation, and data storage, management, reporting and analysis |  |
|  | 2) Work infrastructure | The organization of tasks and responsibilities, within and between individuals and teams, as well as general staffing levels |  |
|  | Communications | The existence of high quality formal and informal information sharing practices within and across structural and professional inner setting boundaries | Method (e.g., conversations, listservs, emails) and type of communication within RWCs (e.g., direct/indirect, among staff, with clients), including documentation of client encounters (e.g. notes in the electronic medical record system) |
|  | Available resources | Resources that are available to deliver and implement the intervention | Available resources for implementation or expansion of the intervention, as well as RWC operations, including clinic assets like mental health professionals, existing mental health programs/services, CABs, physical space, time, etc.  Note: Some aspects of materials & equipment are captured within the operationalization of access to knowledge & information |
|  | 1) Funding | Funding that is available to implement and deliver the innovation |  |
|  | 2) Space | Physical space that is available to implement and deliver the innovation |  |
|  | 3) Materials & equipment | Supplies that are available to implement and deliver the innovation |  |
|  | Access to knowledge & information | Guidance and/or training that is accessible to implement and deliver the innovation | Ease of access to knowledge and information about the intervention and its integration, including access to subject matter experts, training, and online databases and resources |
|  | Recipient-centeredness | The existence of shared values, beliefs, and norms around caring, supporting, and addressing the needs and welfare of recipients | Shared values, beliefs, and norms around caring, supporting, and addressing the needs and welfare of intervention recipients, as well as awareness of and efforts to cater to their needs |
| Individuals/roles | Innovation deliverers | Individuals who are directly or indirectly delivering the innovation | Characteristics of peers delivering the intervention, including responsibilities and desired traits (e.g., knowledge/beliefs/attitudes, compassion/empathy, behavior, personality, lived experiences), as well as needs for engagement |
|  | Innovation recipients | Individuals who are directly or indirectly receiving the innovation | Characteristics of RWC clients who would be receiving the intervention, as well as needs for engagement |
| Individuals/characteristics | Need | Needs related to survival, well-being, or personal fulfillment, which will be addressed by the implementation and/or delivery of the innovation | Personal challenges experienced by RWC clients related to their survival, well-being, or personal fulfillment, which will be addressed by implementation and/or delivery of the intervention, including barriers to access and utilizing MHPSS services |
|  | Capability | The possession of interpersonal competence, knowledge, and skills to fulfill the role | The ability of peers to deliver the MHPSS intervention (e.g., they have interpersonal competence, knowledge, and skills to fulfill the role), including relevant qualifications/credentials (e.g., degrees, training, work experience, lived experience) |
| Process | Planning | Identification of roles and responsibilities, delineation of specific steps and milestones, and definition of goals and measures for implementation success in advance | The extent to which an approach to implementing, monitoring, and evaluating the intervention has been developed in advance (e.g., activities, tasks, methods)  Note: Some aspects of planning were captured within the operationalization of individuals/roles |
|  | Engaging | Efforts to attract and encourage participation in implementation and/or the innovation | Extent to which the organization has attracted and encouraged participation in implementation and/or the innovation (e.g., implementation leads, peers, clients receiving the intervention) |
|  | 1) Innovation deliverers | Efforts to attract and encourage deliverers to serve on the implementation team and/or participate in the innovation | (See definition for Individuals/roles) |
|  | 2) Innovation recipients | Efforts to attract and encourage recipients to serve on the implementation team and/or participate in the innovation | (See definition for Individuals/roles) |
|  | Doing | Implementation in small steps, tests, or cycles of change to trial and cumulatively optimize delivery of the innovation | The extent the intervention is being implemented and completion of implementation as planned (i.e., fidelity) |

Note: *Construct and subconstruct definitions are paraphrased from Damschroder et al. (35) ** The codebook was operationalized using original CFIR definitions from Damschroder et al. (28) but findings were later mapped to CFIR 2.0 definitions.
